# Increased choroidal thickness in adults with Down syndrome

**DOI:** 10.1002/dad2.12170

**Published:** 2021-03-17

**Authors:** Lajos Csincsik, Rachel Nelson, Madeleine J. Walpert, Tunde Peto, Anthony Holland, Imre Lengyel

**Affiliations:** ^1^ Wellcome‐Wolfson Institute for Experimental Medicine Queen's University Belfast Belfast UK; ^2^ Department of Psychiatry University of Cambridge, Cambridge Intellectual and Developmental Disabilities Research Group Cambridge UK

**Keywords:** Alzheimer's disease, biomarker, choroid, dementia, Down syndrome, enhanced depth optical coherence tomography, posterior pole imaging

## Abstract

**Introduction:**

People with Down syndrome (DS) are particularly susceptible to Alzheimer's disease (AD) due to the triplication of the amyloid precursor protein (*APP*) gene. In this cross‐sectional study, we hypothesized that choroidal thinning reported in sporadic AD (sAD) is mirrored in adults with DS.

**Methods:**

The posterior pole of the eye for 24 adults with DS and 16 age‐matched controls (Ctrl) were imaged with optical coherence tomography. Choroidal thickness (ChT) was measured and analyzed in relation to cognitive status and cerebral amyloid beta (Aβ) load.

**Results:**

ChT was increased in people with DS (pwDS) compared to Ctrl. This increase was associated with gender differences and positively correlated with cerebral Aβ load in a small subset. There was no significant correlation detected between ChT and age or cognitive status.

**Discussion:**

In contrast to sAD this study found a significantly thicker choroid in pwDS. Whether these changes are related to Aβ pathology in DS needs further investigation.

## 1 INTRODUCTION

People with Down syndrome (pwDS) are known to be at increased risk of developing Alzheimer's disease (AD) due to the trisomy of chromosome 21, which results in the overproduction of amyloid beta (Aβ) protein and plaque formation in the brain.^1^ Improved support has led to a two‐fold increase in life expectancy for pwDS resulting in AD becoming an increasing concern.^2^

Because the DS population represents the largest cohort of genetic AD, with about 6 million people worldwide, understanding early AD biomarkers in DS is crucial for clinical studies for DS and sAD.^3^, ^4^ An increasing number of studies investigating the brain, cerebrospinal fluid (CSF) and blood biomarkers have shown that the natural history of AD in pwDS is very similar to those with sporadic AD (sAD)^3^, ^5^, ^6^, ^7^, ^8^

As a potential early biomarker for AD, retinal thinning has been investigated in DS. In contrast to what has been observed in sAD or mild cognitive impairment (MCI), the retina was found to be thicker in those with DS compared to age‐matched controls^9^ despite the AD pathology and the accelerated aging effect in DS.^2^, ^10^

In sAD, the choroid also undergoes thinning, both in MCI^11^ and later stages,^12^, ^13^ as assessed in vivo using enhanced depth imaging optical coherence tomography (EDI‐OCT). It is believed that Aβ accumulation in the choroid leads to inflammation, which results in neurodegeneration and vascular attenuation, mirroring the evolution of the amyloid cascade in the brain.^11^, ^13^ Despite the choroid having been proposed as a potential early and non‐invasive biomarker that may reflect neurodegeneration in the brain, the clinical utility of imaging the choroid remains inconclusive in sAD.^14^, ^15^ Given that pwDS overproduce Aβ, studying choroidal thickness (ChT) in adults with DS before or during the onset of clinical dementia, may provide a better insight into early choroidal changes due to developing AD pathology. The only published study in the literature assessed ChT in DS in children and adolescents and found no evidence of choroidal thinning relative to an age‐matched Ctrl group.^16^ However, ChT has never been assessed in adults with DS. For the first time, this cross‐sectional study investigated whether choroidal thinning reported in sAD and MCI is mirrored in adults with DS before clinical evidence of dementia is apparent.

## 2 METHODS

### 2.1 Study recruitment and imaging

The imaging was undertaken by trained examiners in Cambridge, UK. The pwDS were recruited from an existing cohort.^9^ Age‐matched controls were recruited locally in Cambridge. The study was conducted with ethical approval from the East of England Cambridge Central Research Ethics Committee (study ref. ^14^ /EE/1118), and in accordance with the World Medical Association Declaration of Helsinki. Written informed consent was obtained from all individuals, except for those pwDS who lacked the capacity to consent in which case advice was sought from an identified consultee in keeping with the Mental Capacity Act 2005.

**FIGURE 1:**
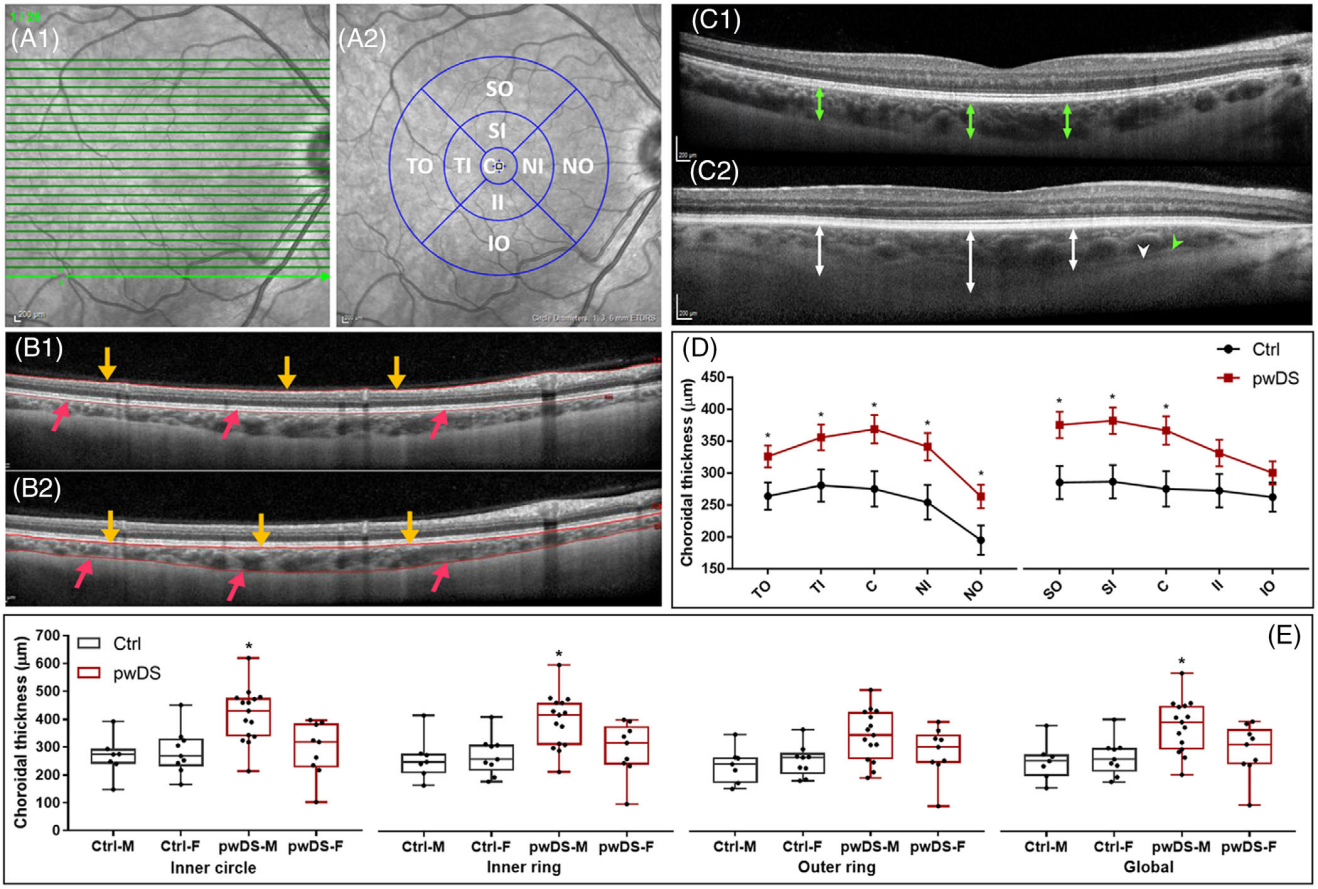
Determination of choroidal thickness in control and in people with Down syndrome. A1 and A2 show the infrared fundus image with green lines demarcating the scanning area (A1. One of the 25 corresponding Enhanced depth imaging (EDI) Optical coherence tomography (OCT) B‐scans (1/25) is depicted in B1 and B2. The retina is outlined as a result of automated retinal segmentation (B1), and the choroid is outlined as a result of manual segmentation (B2). The lines denoting the internal limiting membrane (ILM) (yellow arrows) and Bruch's membrane (BM) (pink arrows) (B1) were moved from their automated position to delineate the retinal pigment epithelium/Bruch's membrane interface and the choroid‐sclera interface respectively (B2). After choroidal segmentation, the thickness values for each sector of the foveal centered Early Treatment Diabetic Retinopathy Study (ETDRS) grid were extracted (A2) and plotted on D (Ctrl = black; pwDS = red). EDI1 OCT2 B scan of the same area of the posterior pole from a Ctrl (C1) and pwDS (C2), illustrating the suprachoroidal layer (SCL) with the hyperreflective stroma (green arrowhead) and the suprachoroidal space (SCS) (white arrowhead). The line graph on D shows the choroidal thickness in Ctrl (black line and error bars) and pwDS (red line and error bars). Estimated marginal mean values of the adjusted model were plotted in each group for each sector of the ETDRS3 grid. Box and whisker plots on E show the unadjusted individual choroidal thickness values in males and females for both Ctrl and pwDS in the inner circle (C), inner ring (average of SI, TI, II, and NI sectors) and outer ring (average of SO, TO, IO, NO sectors) of the ETDRS grid as well as globally (all ETDRS sectors averaged). Abbreviations: Ctrl, control; pwDS, people with Down syndrome; SO, superior outer; SI, superior inner; TO, temporal outer; TI, temporal inner; IO, inferior outer; II, inferior inner; NO, nasal outer; NI, nasal inner; C, inner circle. *, *P* < 0.05

After pharmacological mydriasis (1% tropicamide), all participants were imaged using the Heidelberg Spectralis OCT (Heidelberg Engineering GmbH, Heidelberg, Germany, Camera Model S3610) in EDI mode, between November 2018 and October 2019. Each foveal‐centered EDI scan consisted of 25 high resolution (1536 A scans) 9 times averaged B scans, spaced 240 μm apart, with a total scanned area of 30⁰ × 20°, using Automatic Real‐Time Tracking (ART) and the Retina module (Figure 1 A1).

### 2.2 Choroidal segmentation

As described by Chhablani et al., manual segmentation was performed on each of the 25 horizontal B‐scans for the right eye only of each participant using Heidelberg Eye Explorer (v.1.10.4.0).^17^ The Internal Limiting Membrane (ILM) segmentation line was moved to the RPE/Bruch's membrane interface, and the Bruch's membrane segmentation line to the inner border of the sclera–the choroidal scleral interface (CSI) (Figure 1 B1 and B2). Consequently, the lines now demarcated choroidal rather than retinal thickness. In cases where the suprachoroidal layer (SCL) (hyperreflective suprachoroidal stroma and hyporeflective suprachoroidal space [SCS]) was present, the inner border of the sclera was posterior to the SCS (Figure 1 C2). If the SCL was absent, the inner border of the sclera corresponded to the interface between the hyporeflective vasculature and the hyperreflective sclera (Figure 1 C1). After segmentation, the averaged thickness values of the choroid were manually recorded in micrometers for each sector of the Early Treatment Diabetic Retinopathy Study (ETDRS) grid with circle diameters of 1, 3, and 6 mm (Figure 1 A2). A second grader re‐segmented the single B‐scan crossing the fovea for each study participant to validate the manual segmentation. After re‐segmentation, 13 measurement points were defined, one at the foveola and 500 μm intervals from the foveola to 3 mm nasal and 3 mm temporal (ETDRS grid size). The thickness values for each pre‐defined point were manually recorded, and interobserver agreement was calculated. In addition, retinal thickness values were also extracted using the corresponding posterior pole scans and ETDRS grid, as reported in,^9^ to assess its relationship with ChT.

**HIGHLIGHTS**

- This is the first study investigating ChT1 in pwDS2.
- Manual segmentation revealed an increased ChT_1_ in pwDS_2_.
- There is an increased prevalence of suprachoroidal space in pwDS2.
- Cerebral Aβ3 load correlates with ChT_1_ in a small subset of pwDS_2_.
- The choroidal thickening in pwDS_2_ is contrary to the thinning reported in sAD_4_.

**RESEARCH IN CONTEXT**

1. **Systematic review**: Based on reviewing the available literature, using keywords, optical coherence tomography, choroidal thickness (ChT), and Down syndrome (DS), we identified one study assessing ChT in DS. This study recruited children with DS and found no significant difference in ChT compared to age‐matched controls. This is appropriately cited in our manuscript.
2. **Interpretation**: In contrast to the thinning observed in sporadic Alzheimer's disease (AD), this study found a thicker choroid in adults with DS, before the onset of clinical dementia. The lack of changes in ChT in children with DS suggesting that the thicker choroid detected in our study is a feature of an older DS population and may be the result of developing AD pathology.
3. **Future direction**: As this is the first time that thickening of the choroid is recorded in DS, further studies are needed to verify this observation. Furthermore, it will be important to determine whether the result is due to the developing AD pathology.

EDI scans with missing B scans or missing parts of the CSI, with a quality score (QS) <15 and with scan focus <−6D or >6D were excluded, according to obvious problems (O), poor signal strength (S), centration of scan (C), algorithm failure (A), retinal pathology other than MS related (R), illumination (I) and beam placement (B) (OSCAR‐IB) criteria.^18^ Additional exclusion criteria were history of eye surgery within 3 months of retinal imaging, intravitreal injection, severe cataract, glaucoma, age‐related macular degeneration, and diabetes mellitus. Presence of psychiatric illness other than dementia for the DS group, or including dementia for the control group, was a further exclusion criterion.

#### 2.2.1 CAMCOG‐DS and CAMDEX‐DS

The Cambridge Cognition Examination DS (CAMCOG‐DS) was used to assess areas of cognition known to decline with the onset of dementia.^19^ The Cambridge Examination for Mental Disorders of Older People with Down Syndrome and Others with Intellectual Disabilities (CAMDEX‐DS) was used to assess changes related to the onset of dementia retrospectively and to exclude the possibility of other disorders that mimic dementia.^20^ PwDS did not show signs of clinical dementia based on their CAMCOG‐DS score and CMDEX‐DS assessment. CAMCOG‐DS scores were available for 21 of 24 DS participants.

#### 2.2.2 Brain imaging

For a sub‐set of pwDS, positron emission tomography (PET) scans were acquired in three‐dimensional mode (3D) mode on a General Electric Medical Systems Advanced PET Scanner using Pittsburgh compound^11C^ (PIB). Mean cortical Aβ load was calculated in all cortical regions using the non‐displaceable binding potential (BPND).

### 2.3 Statistical analysis

All analysis was conducted using SPPS (version 26.0; SPSS Inc., Chicago, IL) and data were visualized using GraphPad Prism (version 7). When assessing study characteristics, the chi‐square test was used for categorical variables and independent sample *t* test (with Levene's Test for Equality of Variances) for continuous variables. The continuous variable of ChT was normally distributed as verified by the Shapiro‐Wilk test. Multivariate linear regression adjusted for multiple comparisons (Bonferroni) was used to assess the relationships between ChT of different sectors of the ETDRS grid and diagnosis (Ctrl, DS), with Ctrl as a reference group. Similarly, multivariate linear regression was used to assess the effect of gender on ChT within Ctrl and DS group. The time the retinal scans were acquired was recorded as a categorical variable (AM or PM) and included as a covariate in the final regression model to address diurnal changes in ChT. The right eye was selected as the study eye, and only data from this eye were included in the final analysis. Symmetricity between the right and the left eyes was assessed by paired sample *t* test on fovea‐centered single B‐scans from both eyes.

All *P* values < 0.05 were considered significant. Inter‐observer agreement between the two graders was assessed by calculating intraclass correlation coefficient (ICC). Pearson correlation was used to evaluate the relationship between ChT and age, CAMCOG‐DS scores, brain Aβ load, and retinal thickness.

## 3 RESULTS

### 3.1 Cohort characteristics

There was no significant difference in age or gender ratio between DS and Ctrl groups (Table 1). The mean CAMCOG‐DS score was 78.43, with a standard deviation of ± 16.08 (Table 1).

**TABLE 1:** Study characteristics

Characteristics	Controls N = 16	pwDS N = 24	*P*
Age: y (mean [SD])	36.31 (9.31)	38.50 (7.25)	.410^*^
CAMCOG‐DS^‡^ (mean [SD])	N/A	78.43 (16.08)	N/A
MOF ctx^§^ (mean [SD])	N/A	0.287 (0.08)	N/A
RMF ctx^§^ (mean [SD])	N/A	0.311 (0.05)	N/A
Sex; male [N(%])	7 (43.8)	15 (62.5)	.243^†^
Imaging time; AM (N[%])	11 (68.8)	6 (25.0)	**.006** ^†^
SCS; present (N[%])	0 (0%)	14 (58.3%)	**.000** ^†^

Characteristics for control and pwDS including between‐group comparisons.

Bold numbers indicate a significant difference (*P* < 0.05).

Abbreviations: pwDS, people with Down syndrome; CAMCOG‐DS, Cambridge Cognition Examination DS; AM, ante meridiem; SCS, suprachoroidal space; MOF, medial orbitofrontal; RMF, rostral middle frontal; Ctx, cortex.

^*^Independent sample *t* test for continuous variables; ^†^χ2 test for categorical variable; ^‡^CAMCOG‐DS scores were available for only 21 of 24 pwDS; ^§^Medial orbitofrontal (MOF) and rostral middle frontal (RMF) cortex (ctx) Aβ scores were available for only 6 of 24 pwDS.

### 3.2 There are significant changes in the choroid in pwDS

To assess the reliability of the manual segmentation, inter‐rater agreement was calculated using ICC and was found to range from 0.88 to 0.97, falling in the good‐excellent correlation range. ChT values were not significantly (*p* > 0.05) different between the right and left eyes (Figure S1).

In pwDS, regional differences in ChT across the posterior pole were very similar to the pattern of regional differences measured in the Ctrl group (Figure 1 D). The choroid was thinnest in the nasal outer (NO) and thickest in the superior inner (SI) sectors in both pwDS and Ctrls (Figure 1 D and Table S1). When ChT values were compared between the two groups, apart from the inferior inner (II) sector, a significantly increased ChT was detected across all the sectors of the ETDRS grid in pwDS compared to the Ctrls (Table S1). In the final adjusted model, the differences remained significant in all sectors except in II and IO (Figure 1 D and Table S1).

When gender differences were assessed, a significantly (*P* < 0.05) thicker choroid was found in males compared to females in the inner circle (C; M: 414.60 ± 98.02 μm vs F: 292.11 ± 97.07 μm), the inner ring (average of SI, TI, II and NI sectors; M: 394.61 ± 97.25 μm vs F: 293.38 ± 96.13 μm), and globally (M: 378.14 ± 94.11 μm vs F: 288.63 ± 93.64 μm) in pwDS (Figure 1E and Table S2). Significant differences were not observed in Ctrl (*P* > 0.05) (Figure 1E and Table S2).

The SCS (a hyporeflective band in the SCL) was visible in 14 DS participants (58.3%) but in none of the controls (Table 1 and Figure 1 C2). Those with visible SCS were significantly older (41.6 ± 6.7 years, *P* = .019) than those in whom the SCS was not visible (35.4 ± 8 years). The visibility of the SCS was not associated with increased ChT in DS (Global ChT, SCS not present: 349.9 ± 128 vs SCS present: 340.7 ± 83.7, *P* = 0.843).

### 3.3 There is a correlation between ChT and cerebral Aβ load

**FIGURE 2:**
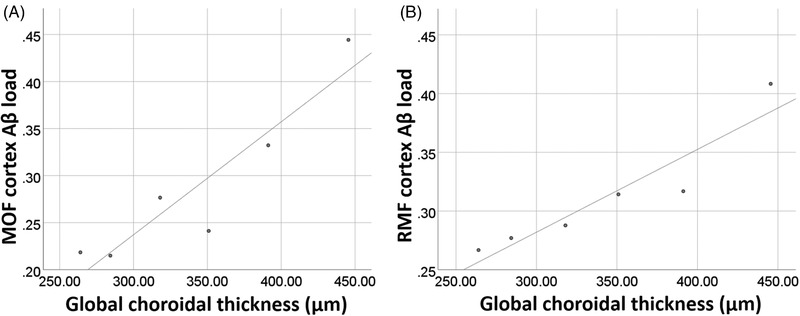
Relationship between choroidal thickness and cerebral Aβ load. The scatter plots show the global choroidal thickness correlation with medial orbitofrontal (A) and rostral middle‐frontal (B) cortex Aβ load. Unadjusted values are plotted, all cortical areas were analyzed, but only the above two showed significant correlation. Abbreviations: MOF, medial orbitofrontal; RMF, rostral middle‐frontal; Aβ, amyloid beta

There was no significant correlation (*P* > 0.05) detected between ChT and age or CAMCOG‐DS (Table 2). Although PET data were only available for a subset of pwDS (n = 6, all male), Aβ load in the medial orbitofrontal (MOF) and the rostral middle‐frontal (RMF) cortex showed a positive correlation with all the sectors of the ETDRS grid except for the SO, SI, and IO (MOF: r = 0.840 to 0.966 and RMF: r = 0.831 to 0.957) (Table 2). Figure 2 illustrates global ChT plotted against Aβ load of the MOF and the RMF cortex. The global ChT remained significant (*P* < 0.05) when the correlation was adjusted for the time of the scan (AM/PM) and/or age (data not presented). A significant correlation between ChT and Aβ load in other parts of the brain could not be detected (data not shown).

**TABLE 2:** The relationship between choroidal thickness and age, cognition, and brain Aβ

	Ctrl, n = 16 r (p)	pwDS, n = 24 r (p)	pwDS, n = 21 r (p)	pwDS, n = 6 r (p)	pwDS, n = 6 r (p)
ChT	Age		CAMCOG	Aβ MOF ctx	Aβ RMF ctx
Superior Outer	−.034 (.902)	−.130 (.545)	.304 (.181)	.778 (.069)	.786 (.064)
Temporal Outer	.300 (.258)	−.255 (.230)	.186 (.419)	**.966 (.002)**	**.957 (.003)**
Inferior Outer	−.041 (.882)	−.227 (.286)	.062 (.791)	.740 (.093)	.773 (.071)
Nasal Outer	−.225 (.402)	−.343 (.101)	.095 (.683)	**.840 (.036)**	**.831 (.040)**
Superior Inner	−.046 (.866)	−.222 (.297)	.371 (.098)	.648 (.164)	.730 (.100)
Temporal Inner	.216 (.422)	−.216 (.310)	.326 (.149)	**.897 (.015)**	**.861 (.028)**
Inferior Inner	−.033 (.905)	−.251 (.237)	.249 (.277)	**.880 (.021)**	**.928 (.008)**
Nasal Inner	−.045 (.868)	−.330 (.115)	.227 (.322)	**.925 (.008)**	**.939 (.005)**
Inner Circle	.187 (.489)	−.293 (.165)	.308 (.174)	**.913 (.011)**	**.902 (.014)**
Centre (SF)	.261 (.329)	−.271 (.200)	.319 (.158)	**.903 (.014)**	**.855 (.030)**
Global	.066 (.808)	−.264 (.212)	.261 (.253)	**.927 (.008)**	**.936 (.006)**

Assessing the relationships between the choroidal thickness of different sectors of the early treatment diabetic retinopathy study (ETDRS) grid and age, Cambridge Cognition Examination DS (CAMCOG‐DS), medial orbitofrontal (MOF), and rostral middle frontal (RMF) cortex (ctx) Aβ load. *P* values were calculated using the Pearson correlation. Bold numbers indicate a significant correlation (*P* < 0.05). Abbreviations: ChT, choroidal thickness; Ctrl, control; pwDS, people with Down syndrome; r, Pearson correlation coefficient; *P*, *P*‐value; SF, subfoveal; MOF, medial orbitofrontal; RMF, rostral middle frontal; ctx, cortex; Aβ, amyloid beta.

In addition, the relationship between ChT and retinal thickness, published earlier,^9^ was also assessed, and found no significant correlation in any of the ETDRS grid sectors (*P* > 0.05) (data not presented).

## 4 DISCUSSION

Identifying early, non‐invasive and inexpensive biomarkers for proxy outcome measure is crucial for the success of AD trials, especially for those pwDS, as many are likely to benefit from treatment trials aimed at preventing the onset of AD pathology. Choroidal thinning has been proposed as an early biomarker for AD in the typically developing population.^11^, ^12^, ^13^ We conducted a detailed choroidal assessment by extracting thickness values for each sector of the ETDRS grid. The thicker choroid detected in our DS cohort with no signs of clinical dementia does not mirror choroidal changes demonstrated in sAD and MCI.^11^, ^12^, ^13^

Choroidal thickening due to increased vessel number in the choroidal stroma was found in a post‐mortem histological study of sAD.^14^ The vascular proliferation could be the response to a metabolic dysfunction of the retina due to retinal Aβ deposition.^21^, ^22^

An increasing number of publications on post‐mortem tissues show Aβ plaque‐like deposits in the retina of donors with sAD.^23^, ^24^, ^25^, ^26^, ^27^ However, not all groups can verify these findings^28^, ^29^, ^30^ or can distinguish between people with sAD and age‐matched, cognitively normal controls.^31^

Only one study examined the presence of Aβ plaques in the retina of pwDS using a modified laser scanning ophthalmoscope and curcumin labeling.^32^ In the absence of controls, this study could not determine the power of the method. Based on emerging data on sAD,^24^, ^33^ the need for further examination of retinal Aβ in vivo is warranted.

We also investigated whether the increased ChT measured in the DS group could result from the SCS that was exclusively visible in pwDS. We found no significant difference in ChT between those with or without SCS. The SCS appears to be an age‐related phenomenon most likely due to fluid accumulation at the scleral and choroidal interface.^34^ It is present in approximately 50% of typically developing individuals above the age of 50 and is associated with hyperopia.^34^ The known accelerated aging effect and the higher prevalence of refractive error (in particular, hyperopia) in pwDS may explain the exclusive presence of SCS in the DS group.^10^, ^35^

ChT is decreased in patients with systemic arterial hypertension and may result from vascular contraction caused by high intravascular pressure in the choroid.^36^, ^37^ A study on healthy volunteers aged 18 and 60 found an inverse relationship between systemic blood pressure and sub‐foveal ChT.^38^ Although blood pressure data were not available for our cohort, DS is associated with lower systemic blood pressure.^39^, ^40^ Therefore, we speculate that the increased ChT observed in our DS group might be, at least partially, due to lower systemic and or choroidal blood pressure.

We have previously shown that a thicker retina was associated with DS, and the thickening occurred in the inner retinal layers.^9^ pwDS have a markedly increased number of vessels in the retinal, which is believed to be the consequence of altered angiogenesis in DS.^41^ Because retinal vessels are present in the inner retinal layers, the higher vessel number may contribute to the inner retinal layer changes.^41^ Therefore, apart from the decreased blood pressure, the altered angiogenesis in pwDS may also contributor to the increased ChT.

A recent study showed no significant increase in ChT in children with DS,^16^ suggesting that the significantly thicker choroid detected in our study is a feature of an older DS population. This raises the question of whether the observed choroidal thickening in pwDS could result from developing AD pathology. The positive correlation between ChT and the increased Aβ load in the MOF and RMS cortices could be related to inflammatory changes observed in early AD stages.^42^ It is perhaps not surprising that differences are associated with the frontal cortices, as these areas are among the first that are affected by Aβ deposition in pwDS.^4^ However, the results should not be overinterpreted, considering the low number of study participants with PET data in this study and the lack of correlation with other primarily affected areas such as the striatum.^4^

While information on choroidal changes are sparse,^15^ retinal thickening had been reported in early stages of sporadic AD (preclinical AD and MCI), and believed to be the result of inflammatory processes due to AD pathology.^43^, ^44^ Hence, we cannot rule out the possibility that the thickened choroid in our cohort is, at least partially, the result of inflammatory processes due to developing AD pathology.

We found thinner choroid in females compared to males in pwDS, a difference that was not detected in the Ctrl group. The same heterogeneity in ChT between females and males has been observed in the general population,^45^ and it is believed to be driven by hormonal changes in postmenopausal women.^46^ Although the information on the onset of menopause in our study was not available, it is well known that the menopause occurs earlier in pwDS women than the general population and is associated with an increased risk of dementia.^47^ Phenotypic variability has been shown in sAD, and gender was a significant driving force.^48^ We are not aware of studies reporting gender‐related phenotypic differences in choroidal or retinal thickness in pwDS or sAD. Future studies assessing eye biomarkers for AD should consider gender in study design based on our observation.

Decreased ChT had been associated with age.^49^ However, we did not observe a significant age effect on ChT in the Ctrl or pwDS, probably due to the relatively young age and the limited age‐range in our study. Previous studies have also demonstrated a positive correlation between ChT and cognitive status in sAD and MCI,^11^ but this was probably not detectable in our cohort due to the limited differences in cognitive scores. Therefore, further studies should elucidate further this relationship between ChT and cognitive measures in pwDS. Tan et al. demonstrated that there are diurnal variations in ChT but this could also be influenced by refractive error, intraocular pressure (IOP), and blood pressure.^50^ Although our analysis controlled for diurnal variation, refractive error, IOP and blood pressure data were not available. We demonstrated a potential relationship between brain Aβ load and ChT in a subset of pwDS (n = 6). However, further studies will need to verify this relationship due to the small sample size and possible gender bias.

One of our study's strengths was the comprehensive analysis undertaken by segmenting all the 25 OCT B‐scans per posterior EDI scan, which allowed us to extract data for each sector of the ETDRS grid. To ensure that the manual choroidal segmentation was robust, we used a second grader and found an excellent inter‐observer agreement.

Overall, this study reports for the first time choroidal thickening in adults with DS with no signs of clinical dementia. Further studies are needed with additional brain imaging components to elucidate whether the observed choroidal thickening in pwDS results from developing AD pathology and/or a developmental aspect of DS. Understanding the cellular and molecular changes underpinning this thickening will help gain better insight into early AD‐related pathological changes in pwDS. Imaging the choroid may provide a new understanding of how to detect or monitor disease progression and assess disease‐modifying interventions.

## CONFLICTS OF INTEREST

The authors declare no conflict of interests.

## STATEMENT OF SIGNIFICANCE

This is the first study that reports choroidal thickening in the eye in adults with Down syndrome. This finding is in contrast to the thinning observed in sporadic Alzheimer's disease.

## Supporting information



Supporting Information

## Data Availability

The datasets generated during and/or analysed during the current study are available from the corresponding author on reasonable request.
