# Supplementary material for: Increased choroidal thickness in adults with Down syndrome
Source: Alzheimers Dement (Amst). 2021 Mar 17;13(1):e12170. doi: 10.1002/dad2.12170 (PMC7967920; doi:10.1002/dad2.12170)
Supplement: Supplementary file 1 — Supporting Information [file DAD2-13-e12170-s001.pdf]

Supplementary material:

Table S1

Comparison of choroidal thickness between Ctrl and pwDS

Choroidal thickness (µm)	Ctrl, n=16 Mean (SD)	pwDS, n=24 Mean (SD)	Mean difference (95% CI)	p values
Superior Outer[*]	282.37 (62.77)	377.08 (112.21)	94.70 (32.11 to 157.29)	**.004**
Superior Outer[†]	285.07 (103.48)	375.28 (101.31)	90.20 (19.84 to 160.57)	**.013**
Temporal Outer[*]	263.81 (58.25)	326.54 (89.52)	62.72 (11.32 to 114.13)	**.018**
Temporal Outer[†]	264.64 (85.08)	325.98 (83.33)	61.34 (3.48 to 119.19)	**.038**
Inferior Outer[*]	253.50 (70.00)	306.00 (94.06)	52.50 (-3.28 to 108.28)	.064
Inferior Outer[†]	262.34 (90.80)	300.10 (88.92)	37.75 (-24.00 to 99.51)	.223
Nasal Outer[*]	181.87 (66.24)	271.95 (99.47)	90.08 (32.67 to 147.49)	**.003**
Nasal Outer[†]	194.88 (91.80)	263.28 (89.90)	68.40 (5.96 to 130.84)	**.033**
Superior Inner[*]	284.25 (68.35)	383.20 (110.71)	98.95 (36.07 to 161.84)	**.003**
Superior Inner[†]	286.30 (104.00)	381.84 (101.85)	95.54 (24.80 to 166.28)	**.009**
Temporal Inner[*]	279.18 (70.37)	356.62 (104.67)	77.43 (16.89 to 137.94)	**.014**
Temporal Inner[†]	280.50 (100.16)	355.74 (98.13)	75.24 (7.11 to 143.37)	**.031**
Inferior Inner[*]	261.75 (78.49)	338.20 (108.71)	76.45 (12.48 to 140.42)	**.020**
Inferior Inner[†]	272.44 (103.92)	337.01 (101.80)	58.62 (-12.06 to 129.31)	.101
Nasal Inner[*]	242.87 (82.57)	348.58 (112.62)	105.70 (39.17 to 172.24)	**.003**
Nasal Inner[†]	254.15 (108.04)	341.06 (105.82)	86.90 (13.42 to 160.39)	**.022**
Inner Circle[*]	277.31 (76.12)	368.66 (113.12)	91.35 (25.91 to 156.79)	**.007**
Inner Circle[†]	282.98 (107.80)	364.88 (105.57)	81.90 (8.59 to 155.21)	**.030**
Centre (SF)[*]	271.88 (76.80)	368.88 (116.69)	97.00 (29.82 to 164.17)	**.006**
Centre (SF)[†]	275.24 (111.00)	366.62 (108.71)	91.38 (15.89 to 166.87)	**.019**
Global[*]	259.88 (66.37)	344.57 (101.98)	84.69 (26.12 to 143.25)	**.006**
Global[†]	265.85 (96.28)	340.59 (94.31)	74.73 (9.25 to 140.20)	**.026**

Multivariate liner regression model assessing the relationships between choroidal thickness of different sectors of the ETDRS[1] grid and diagnosis (Ctrl, pwDS) with Ctrl as a reference group, unadjusted and adjusted for AM/PM[2]. Reported means are the estimated marginal means of the adjusted model. Bold numbers indicate a significant difference ($p<0.05$). Rows from the sectors of the ETDRS[1] grid on the horizontal meridian are shaded (grey). Abbreviations: Ctrl, control; pwDS, people with Down's syndrome; CI, confidence interval; SF, subfoveal; SD, standard deviation;

[*]unadjusted; [†]adjusted for AM/PM;

[1] Early Treatment Diabetic Retinopathy Study
[2] Ante meridiem/Post meridiem

Table S2

The relationship between gender and choroidal thickness

Choroidal thickness (µm)	Mean (SD)	Mean (SD)	Mean difference (95% CI)	p values
**Ctrl**	**Male, n=7**	**Female, n=9**		
Inner Circle	267.28 (72.70)	285.11 (82.11)	17.82 (-66.72 to -102.37)	.658
Inner ring	260.89 (78.48)	271.77 (70.50)	10.88 (-69.13 to 90.90)	.775
Outer ring	236.17 (64.67)	252.55 (56.66)	16.37 (-48.72 to 81.47)	.598
Global	251.31 (70.09)	266.54 (66.80)	15.23 (-58.52 to 88.98)	.665
**pwDS**	**Male, n=15**	**Female, n=9**		
Inner Circle	414.60 (98.02)	292.11 (97.07)	-122.48 (-207.90 to -37.07)	**.007**
Inner ring	394.61 (97.25)	293.38 (96.13)	-101.22 (-185.91 to -16.54)	**.021**
Outer ring	343.21 (90.97)	282.36 (89.37)	-60.85 (-139.90 to 18.18)	.125
Global	378.14 (94.11)	288.63 (93.64)	-89.50 (-171.65 to -7.36)	**.034**

Multivariate linear regression model assessing the relationships between choroidal thickness (inner ring (average of SI[3], TI[4], II[5] and NI[6] ETDRS[7] sectors), outer ring (average of SI[8], TI[9], II[10] and NI[11] ETDRS sectors) and global (all ETDRS sectors averaged)) and males as a reference group in Ctrl and pwDS. Bold numbers indicate a significant difference (p<0.05). Abbreviations: Ctrl, control; pwDS, people with Down's syndrome; CI, confidence interval; SD, standard deviation;

[3] Superior inner
[4] Temporal inner
[5] Inferior inner
[6] Nasal inner
[7] Early Treatment Diabetic Retinopathy Study
[8] Superior outer
[9] Temporal outer
[10] Inferior outer
[11] Nasal outer

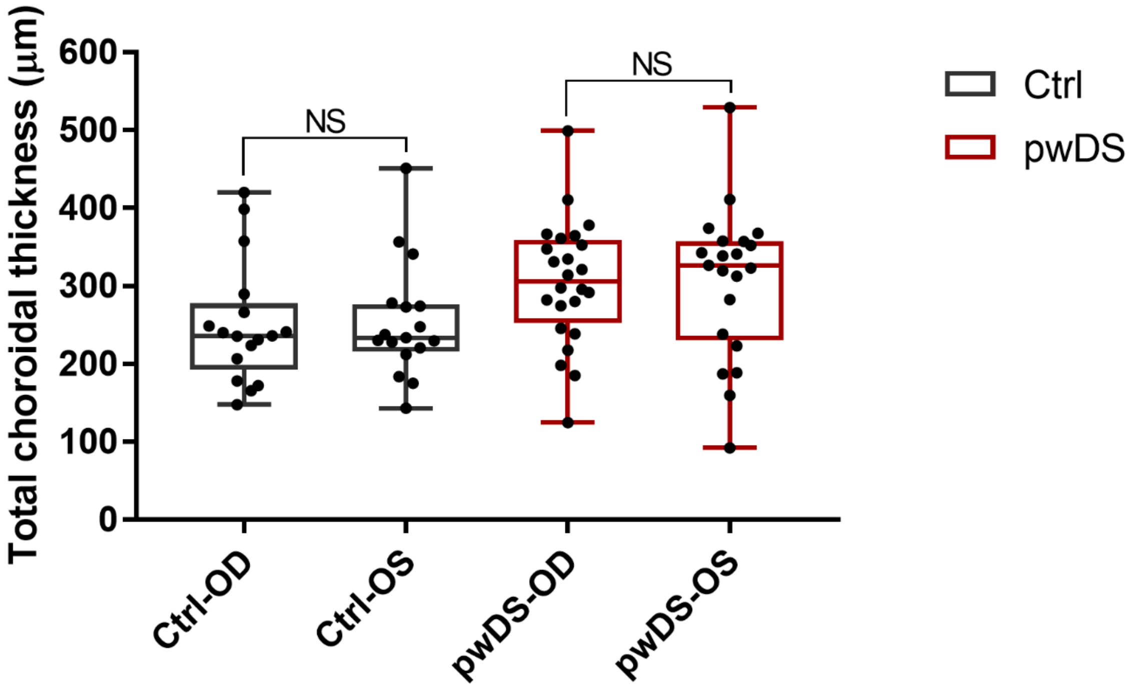

Figure S1 Choroidal thickness in the right and the left eyes

Box and whisker plots show the unadjusted individual choroidal thickness values in the right (OD) and left (OS) eyes in control (black) and people with Down's syndrome (red). On a single B-scan crossing the fovea 13 measurement points were defined, one at the foveola and 500 μm intervals from the foveola to 3 mm nasal and 3 mm temporal (ETDRS grid size). The thickness values for each pre-defined point were manually recorded and then averaged to generate total choroidal thickness. P values were calculated using paired sample T-test. Significance level was set to 0.05. Abbreviations: Ctrl, control; pwDS, people with Down's syndrome; OD, oculus dextra (right eye); OS, oculus sinistra (left eye); NS, none-significant;
